# Apolipoprotein E deletion has no effect on copper-induced oxidative stress in the mice brain

**DOI:** 10.1042/BSR20180719

**Published:** 2018-09-07

**Authors:** Yuan Chen, Liang Wang, Jiang-hui Geng, Hui-feng Zhang, Li Guo

**Affiliations:** 1 Department of Pediatrics, The Second Hospital of Hebei Medical University, Shijiazhuang, Hebei 050000, China; 2 Key Laboratory of Hebei Neurology, Shijiazhuang, Hebei 050000, China; 3 Department of Neurology, The Second Hospital of Hebei Medical University, Shijiazhuang, Hebei 050000, China; 4 Department of Medicine, The First Hospital of Shijiazhuang, Shijiazhuang, Hebei 050011, China

**Keywords:** ApoE knockout mice, copper buildup, oxidative stress, Wilson’s disease

## Abstract

The current study was designed to investigate effect of copper administration on oxidative damage to the brain in *ApoE*^−/−^ mice and to explore the putative neuroprotective effects rendered by apolipoprotein E (ApoE). Male C57BL/6 *ApoE*^−/−^ and wild-type mice were randomly assigned into four groups, *ApoE^−/*−*^* mice wild-type mice treated with either copper or saline. Copper sulphate pentahydrate or saline (200 µl) were administered intragastrically daily for 12 weeks. Expression of malondialdehyde, superoxide dismutase (SOD), hemeoxygenase 1 (HO-1), and NAD(P)H: quinone oxidoreductase 1 (NQO1) were determined by a combination of biochemical assays. The concentration of copper in the brain of *C57BL/6* mice and *ApoE^−/*−*^* mice treated by copper significantly increased compared with mice treated by saline (*P*=0.0099 and *P*=0.0443). Compared with the *C57BL/6* mice treated by copper, the level of the *ApoE^−^/^−^* mice treated by copper was higher (*P*=0.018). TBARS and SOD activities or the expressions of NQO1 and HO-1 in the brain were not significantly different amongst the four experimental groups of mice. The relative value of NQO1/β-actin expression in the brain of the *ApoE*^−/−^ mice was similar in both saline and copper administration experimental groups. However, Western blot analysis showed that NQO1 expression was significantly higher in the *ApoE^−/−^* mice brain treated with saline compared with saline treated wild-type mice (*P*=0.0449). *ApoE* does not function in protecting the brain from oxidative damage resulting from copper build-up in Wilson’s disease, but may play a role in regulating copper accumulation in the brain.

## Introduction

Hepatolenticular degeneration, also termed as Wilson’s disease is an autosomal recessive genetic disorder and is characterized by *ATP7B* gene mutations, leading to defective copper metabolism [1]. Wilson’s disease occurs approximately amongst 1 in 30000 live births [2]. The H1069Q mutation in European populations and the R778L mutation in Asian populations are the most common *ATP7B* mutation observed in patients with Wilson’s disease [2–5].

Hepatic damage to various extents, neuropsychiatric symptoms, Kayser–Fleischer rings, and damage to the kidney and skeletal muscle are classical clinical hallmarks in Wilson’s disease. Interestingly, liver damage is generally the major clinical manifestation at disease onset in children, whereas neurological symptoms tend to occur in those aged in their twenties or older [6]. There are though some reports of pediatric cases where primary symptoms were neurological, either isolated or along with mild hepatic injury. The diversity of symptoms and differences in age at disease onset suggest that the features of this disease may not be determined exclusively by the *ATP7B* gene mutations and that other genetic factors may be involved in the pathogenesis and perhaps progression of the disease.

Recently, some studies have shown that ages of onset as well as diversity of clinical presentation are correlated to the variant apolipoprotein E (ApoE) genotypes [7–9]. There are three alleles of the *ApoE* gene – ε2, ε3, and ε4 – encoding three different ApoE isoforms, each with biological functions different from the other [10]. Several lines of evidence have proved beyond doubt that the ApoE genotypes are associated with occurrence and clinical outcome of neurodegenerative diseases [10–14]. Whereas the ApoE 3 protein has neuroprotective function, the ApoE 4 protein increases vulnerability to CNS damage [15–17]. Because ApoE proteins can exert an antioxidant effect [18], and high copper build-up in Wilson’s disease induce tissue damage via generation of free oxygen radicals [1], it is speculated that ApoE may render a neuroprotective effect in patients with Wilson’s disease.

We have earlier determined the role of ApoE post copper administration induction of hepatic toxicity by modeling the *ApoE^−/−^* mice. We confirmed that copper accumulation can induce hepatic oxidative damage and that ApoE may protect the liver from this oxidative injury [18]. Given that copper accumulation also occurs in the brain, the objective of the current study was to explore whether ApoE exerts neuroprotective effect against oxidative damage induced by copper accumulation in the brain.

## Methods

### Animals

Animal experiments were performed after obtaining approval from the Institutional Animal Care and Use Committee of Hebei Medical University, Hebei, China, as described before [20]. Male C57BL/6 *ApoE^−/−^* and wild-type mice (15.1 ± 1.38 g; Animal Care Center of Beijing Medical University, China) were used in the present study. Animals were maintained as described before [20]. Twenty-four wild-type or *ApoE^−/−^* mice were randomly assigned into two groups – either treated with saline or copper. Copper sulphate pentahydrate (200 µl; 200 ppm; lot# BCBG7381V, Sigma, U.S.A.) or 200 µl saline was intragastrically administered daily for 12 weeks. After 12 weeks, mice (*n*=8 per group) were killed, and blood was obtained by orbital bleeding. The brains were removed, snap-frozen, and stored in liquid nitrogen until further use. The remaining four mice from each group were processed for immunohistochemistry as described below.

### Preparation of tissue samples

Brain tissue homogenates were prepared using ice-cold PBS containing protease inhibitor cocktail (Sigma–Aldrich, U.S.A.) by homogenizer. Homogenates were centrifuged at 8000 ***g*** for 10 min. TBARS and superoxide dismutase (SOD) activities were determined in the supernatants as described in the next section.

### Determination concentration of copper in brain

The concentration of copper was determined according to the method as described in previous study [20]. Two hundred milligrams of brain samples were digested using 5 ml of concentrated nitric acid and 1 ml of 30% H_2_O_2_ in a microwave digestion system (CEM, U.S.A.). And all samples were heated to 140°C until the volume declined to 2 ml after they cooled down. Each sample was washed for three times using 1% nitric acid. The atomic absorption spectrometer was used to determine the concentration of copper.

### Determination of TBARS and SOD activities in the brain

TBARS activities were determined using the thiobarbituric acid (TBA) method (Nanjing Jiancheng Bioengineering Institute, China) as described before [20]. The concentration of TBARS was counted as nM of TBARS per mg of protein. SOD activities were determined by the xanthine oxidase method as described previously [20], and the results were presented as units per milligram of protein.

### Immunohistochemistry

After copper or saline administration for 12 weeks, four mice were randomly selected from each group for immunohistochemical experiments using SABC three-step kit (Boster, Wuhan, China). Intraperitoneal injection of 10% chloral hydrate (300 mg/kg) was used for anesthesia. Post-anesthesia mice were transcardially perfused with 200 ml of normal saline followed by 4% paraformaldehyde in PBS. The brains were removed and fixed for 24 h in 4% paraformaldehyde, and embedded in paraffin. Tissue sections (5 µm) were obtained using a microtome. Treatment with 3% H_2_O_2_ at room temperature for 15 min was done to block the endogenous peroxidase activity. Sections were then processed as described previously [20] and incubated with primary antibodies against hemeoxygenase 1 (HO-1; 1:100 dilution, Epitomics, U.S.A.) or NAD(P)H: quinone oxidoreductase 1 (NQO1; 1:100 dilution, Epitomics, U.S.A.) overnight at 4°C. Sections were developed using DAB staining and were then counterstained with Hematoxylin. The sections not containing primary antibodies were used as negative controls. A light microscope was used to image the immunostained sections.

### Western blot

Immunoblot analysis of proteins expressed in brain was assayed in lysates made from brain tissues as described before [20].

### Quantitative real-time PCR

Total RNA was isolated with TRIzol reagent (Thermo Fisher Scientific, Shanghai, China) and treated with DNase to get rid of any contaminating genomic DNA. cDNA and quantitative real-time PCR (qRT-PCR) analysis were performed as described before [20]. Primers used were *NQO1* forward primer 5-TATGCTGCCATGTACGACAACGG-3, reverse primer 3- AAGACCTGGAAGCCACAGAAACG-5. Data analysis was performed using the Sequence Detection Systems interface (Thermo Fisher Scientific).

### Statistical analysis

Data were expressed as mean ± S.D. Two-way ANOVA was used for comparing continuous data between male C57BL/6 ApoE^−/−^ group and wild-type mice group that expression of malondialdehyde, SOD, HO-1, and NQO1 data, which were determined by a combination of biochemical assays. If the interaction was significant, least square mean test was used for pairwise comparison only if interaction was statistically significant. All statistical analysis was performed with SPSS 21.0 software and *P*<0.05 was considered as statistically significant.

## Results

### The concentration of copper in the brain of ApoE^−/−^ mice

**Figure 1:**
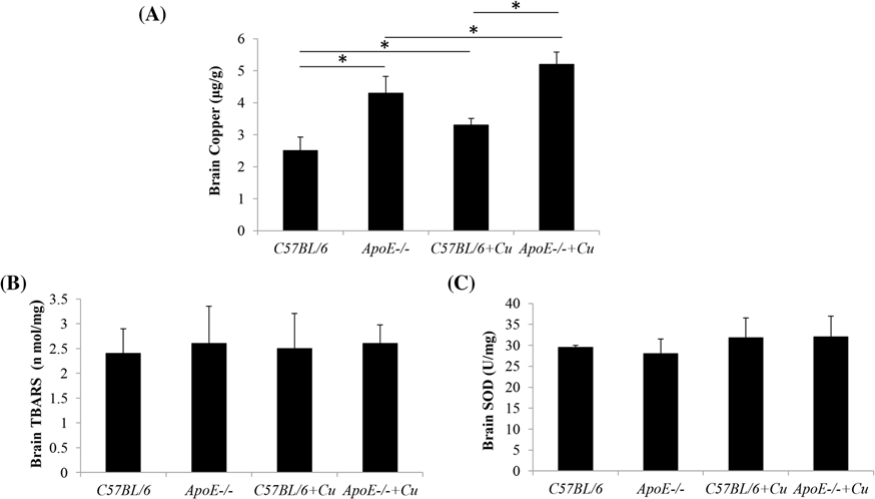
Concentration of copper (A), TBARS (B) and SOD (C) in the brain. (A) The level of brain copper in C57BL/6, *ApoE^−/^*^−^, C57BL/6+Cu, and *ApoE^−/^*^−^+Cu groups. (B) The levels of brain TBARS in the four groups were determined. (**C**) The levels of brain SOD in the C57BL/6 or *ApoE^−/^*^−^ mice after being treated by saline or copper were determined. Data were expressed as mean ± S.D.; *P<0.05.

To verify the concentration of copper in the brain of the four groups, we determined the concentration of copper used the atomic absorption spectrometer. The results showed that *ApoE^−/−^* mice treated with saline exhibited significantly higher copper concentrations in the brain compared with the wild-type mice treated with saline (4.3 ± 0.52 compared with 2.5 ± 0.43; *P*=0.0099). The copper concentration of *C57BL/6* mice after treatment of copper was significantly higher compared with the *C57BL/6* mice treated by saline in the brain tissue (3.3 ± 0.21 compared with 2.5 ± 0.43; *P*=0.0443). In the *ApoE^−/−^* mice, the treatment of copper also increased the copper concentration of *ApoE^−/−^* mice brain (5.2 ± 0.39 compared with 4.3 ± 0.52; *P*=0.039). And compared with the *C57BL/6* mice treated by copper, the level of the *ApoE^−/−^* mice treated by copper was higher (5.2 ± 0.391 compared with 3.3 ± 0.21; *P*=0.018). The results are shown in Figure 1A.

### TBARS and SOD activities in the brain of ApoE^−/−^ mice

We determined the oxidative stress through TBARS and SOD. The results showed that there were no significant differences in brain TBARS and SOD activities amongst the wild-type mice treated with saline or copper (TBARS: C57BL/6, 2.4 ± 0.5 compared with C57BL/6+Cu, 2.5 ± 0.71; SOD: C57BL/6, 29.5 ± 0.5 compared with C57BL/6+Cu, 31.8 ± 4.7; *P*=0.50 and 0.468) and the *ApoE^−/−^* mice treated with saline or copper (TBARS: *ApoE^−/^*^−^, 2.6 ± 0.75 compared with *ApoE^−/−^*+Cu, 2.6 ± 0.38; SOD: *ApoE^−/−^*, 28 ± 3.5 compared with *ApoE^−/−^*+Cu, 32 ± 5; *P*=0.981 and 0.3197) (Figure 1B,C).

### Expression of HO-1 and NQO1 in the brain of ApoE^−/−^ mice

**Figure 2:**
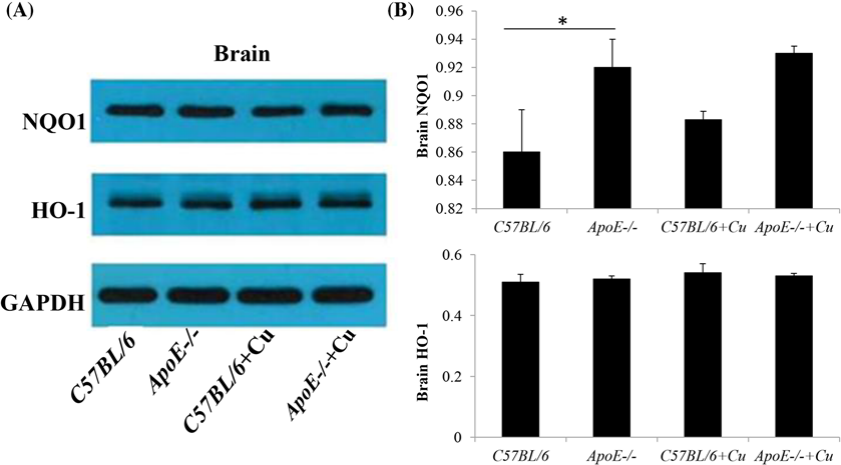
The expression level of NQO1 and HO-1 was determined by Western blot (**A**) The protein expression levels of NQO1 and HO-1 in the brain of in C57BL/6, *ApoE^−/^*^−^, C57BL/6+Cu, and *ApoE^−/^*^−^+Cu groups. (**B**) Results in (A) for all eight mice per group are summarized. Data were expressed as mean ± S.D.; *P<0.05.

**Figure 3:**
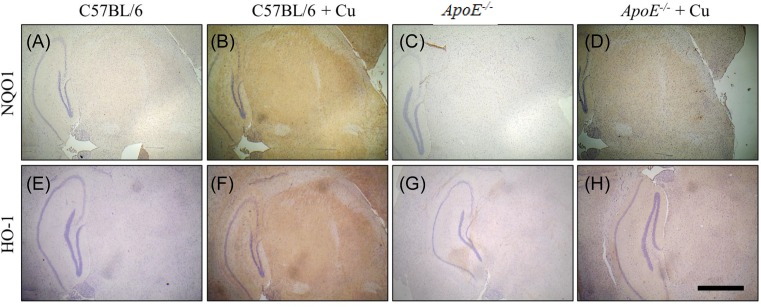
NQO1 and HO-1 expression in the brain were determined by immunohistochemistry (**A**) The NQO1 expression was detected in C57BL/6 group. (**B**) The NQO1 expression was detected in C57BL/6+Cu group. (**C**) The NQO1 expression was detected in *ApoE^−/^*^−^ group. (**D**) The NQO1 expression was detected in *ApoE^−/^*^−^+Cu group. (**E**) The HO-1 expression was detected in C57BL/6 group. (**F**) The HO-1 expression was detected in C57BL/6+Cu group. (**G**) The HO-1 expression was detected in *ApoE^−/^*^−^ group. (H) The HO-1 expression was detected in *ApoE^−/^*^−^+Cu group.

Western blot analysis showed that NQO1 expression was significantly higher in the *ApoE^−/−^* mice brain treated with saline compared with saline treated wild-type mice (0.86 ± 0.03 compared with 0.92 ± 0.02; *P*=0.0449; Figure 2A,B). However, there was no significant difference in NQO1 expression between the saline or copper treated wild-type (0.883 ± 0.006 compared with 0.86 ± 0.03; *P*=0.2541) or *ApoE^−/−^* mice (0.93 ± 0.005 compared with 0.92 ± 0.02; *P*=0.2476) (Figure 2A,B). And then we used immunohistochemistry to detect the expression of NQO1 and HO-1 in the four groups; similar results were obtained (Figure 3A–D). No difference in expression of HO-1 in the saline or copper treated wild-type or *ApoE^−/^*^−^ mice was found (C57BL/6, 0.51 ± 0.025 compared with C57BL/6+Cu, 0.54 ± 0.03 compared with *ApoE^−/−^*, 0.52 ± 0.01 compared with *ApoE^−/−^*+Cu, 0.53 ± 0.008; Figures 2A,B and 3E–H).

### The expression of *NQO1* mRNA in the brain

**Figure 4:**
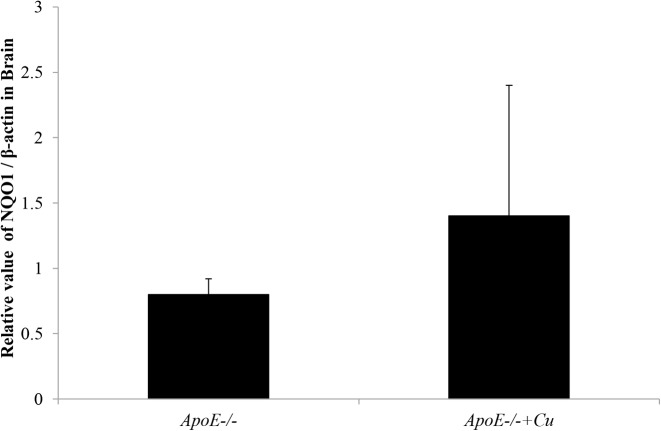
The relative value of NQO1/β-actin in brain in *ApoE^−/*−*^* treated with saline (*ApoE^−/−^*), and *ApoE^−/−^* treated with copper (*ApoE^−/−^*+Cu) Data were expressed as mean ± S.D.

To determine the expression of NQO1, we also used the qRT-PCR to detect the level of NQO1 in ApoE^−/*−*^ mice. Even though the relative ratio of *NQO1* and *ACTB* (encoding β-actin) in the *ApoE^−/−^* mice brain treated with copper was slightly more than the *ApoE^−/−^* mice treated with saline, the difference did not attain statistical significance (1.4 ± 1 compared with 0.8 ± 0.12; *P*=0.3605; Figure 4).

## Discussion

Clinical outcomes in vascular and neurodegenerative disorders seem to be regulated by the *ApoE* genotypes [14,19,20]. In Alzheimer’s disease, the ApoE 3 protein functions as a neuroprotector; in comparison, the ApoE 4 protein promotes CNS injury [15–17]. Several studies have shown that APOE ε4-positive genotype is associated with earlier symptomatic visibility of Wilson’s disease, particularly amongst patients harboring the *ATP7B* p.H1069Q homozygous patients in women [7–9]. However, whether ApoE facilitates neuroprotection in Wilson’s disease was not known.

In an earlier study [20], we showed the role of ApoE in copper buildup-induced oxidative injury in the liver by administering copper for 12 weeks at high dosage of 200 mg/kg/day to ensure effectiveness of copper toxicity. The animals treated with copper exhibited a significantly higher copper concentration in the liver than the animals treated with saline, suggesting that excessive copper accumulates in the liver. Furthermore, copper treatment also resulted in excessive copper accumulation in other organs including the brain and kidney. Thus, this animal model exhibited copper build-up in the brain, kidney, and liver with wide-ranging hepatic damage, which mimics Wilson’s disease presentation features. We found that copper induced an elevation in TBARS activities and an inhibition in SOD activities in the serum and liver respective of ApoE expression, accompanied by an elevation in the expression of NQO1 and HO-1, indicating that elevation of copper concentration can induce oxidative damage in the liver.

Copper functions as the cofactor of various enzymes participating in a diverse array of cellular processes. Therefore, it is likely that pleiotropic mechanisms regulate copper toxicity. High levels of copper were associated with neural degeneration, reducing the number of nerve cells [21], and miniature quantities of copper-induced neurotoxicity in cholesterol-fed mice via oxidative stress-induced apoptosis [22].

In this study, the mice were also exposed to copper administration for 12 weeks with the high dosage of 200 mg/kg/day to ensure effectiveness of copper toxicity. In toxicologic studies, copper is usually administered in doses of 50 mg/kg/day as low toxicity, 100 mg/kg/day as moderate toxicity, and 200 mg/kg/day as high toxicity [23]. On the other hand, it has been reported that, hepatic injury might occur after a period of 12 weeks feeding with standard chow diet [24,25]. In the 12 weeks, the copper accumulated in the brain and the hepatic injury was not very serious. In light of the above, 12 weeks of copper in high dosage was chosen. We found that the *ApoE^−/−^* mice treated with saline exhibited significantly higher copper concentrations in the brain compared with the wild-type mice treated with saline, and the *ApoE^−/−^* mice treated with copper sulphate pentahydrate also exhibited significantly higher copper concentrations in the brain compared with the wild-type mice treated with copper sulphate pentahydrate, suggesting that *ApoE* may participate in regulating copper accumulation in the brain. However, it remains unknown how *ApoE* knockout results in copper accumulation in the brain. The blood–brain barrier is disrupted in *ApoE^−/−^* mice [26] resulting in the possibility that increase in the permeability of the blood–brain barrier to copper may lead to an increase in brain copper concentrations in *ApoE^−/−^* mice. However, though brain copper concentrations were significantly higher in the wild-type mice treated with copper and the *ApoE^−/−^* mice treated with saline or copper compared with the wild-type mice treated with saline, no significant differences in TBARS and SOD activities or the expression of NQO1 and HO-1 were found amongst the saline or copper treated wild-type mice and *ApoE^−/−^* mice.

Even though copper deposited in the liver is thought to initiate Wilson’s disease, the disease presentation does not match with degree of copper accumulation in the liver, indicating that a multitude of factors may contribute significantly to Wilson’s disease pathology [27]. These factors include the exposure time to copper, the intracellular copper distribution, the presenting form of copper in the liver, and the involvement of additional protein regulators. Due to the expression of *ATP7B* in the CNS, the CNS abnormalities may be accompanied by liver damage, and the delay of neurological symptoms may be arising from partial compensation of *ATP7B* malfunction by another copper-transporting ATPase, ATP7A, which is expressed in the brain, but not in the liver cells.

To further investigate the impact of ApoE on the brain, we detected both the expression of *NQO1* mRNA, and the relative value of NQO1/β-actin in the brain. The results by the two tests were in accordance, showing no differences between the *ApoE^−/−^* mice with either, suggesting that ApoE may not produce neuroprotective roles in copper-induced oxidative damage further.

In summary, our results showed that knockout of *ApoE* potentiated copper buildup-induced oxidative damage in the brain. However, although the brain copper concentrations increased in the *ApoE^−/−^* mice, no significant differences in the TBARS and SOD activities or the expression of NQO1 and HO-1 were found in the copper treated *ApoE^−/−^* mice. Our findings suggest that ApoE may not protect the brain from copper-induced oxidative damage but might play a yet undefined role in regulating copper accumulation in the brain.

## Abbreviations

ApoE: apolipoprotein E
CNS: central nervous system
HO-1: hemeoxygenase 1
NQO1: NAD(P)H: quinone oxidoreductase 1
qRT-PCR: quantitative real-time PCR
SOD: superoxide dismutase
TBARS: thiobarbituric acid reactive substances

## References

[1] Petrukhin K., Lutsenko S., Chernov I., Ross B.M., Kaplan J.H. and Gilliam T.C. (1994) Characterization of the Wilson disease gene encoding a P-type copper transporting ATPase: genomic organization, alternative splicing, and structure/function predictions. Hum. Mol. Genet. 3, 1647–1656 10.1093/hmg/3.9.1647 7833924

[2] Gollan J.L. and Gollan T.J. (1998) Wilson disease in 1998: genetic, diagnostic and therapeutic aspects. J. Hepatol. 28, 28–36 10.1016/S0168-8278(98)80373-5 9575447

[3] de Bie P., Muller P., Wijmenga C. and Klomp L.W. (2007) Molecular pathogenesis of Wilson and Menkes disease: correlation of mutations with molecular defects and disease phenotypes. J. Med. Genet. 44, 673–688 10.1136/jmg.2007.052746 17717039 PMC2752173

[4] Gu Y.H., Kodama H. and Du S.L. (2005) Apolipoprotein E genotype analysis in Chinese Han ethnic children with Wilson’s disease, with a concentration on those homozygous for R778L. Brain Dev. 27, 551–553 10.1016/j.braindev.2005.01.006 16310588

[5] Czlonkowska A., Rodo M., Gajda J., Ploos van Amstel H.K., Juyn J. and Houwen R.H. (1997) Very high frequency of the His1069Gln mutation in Polish Wilson disease patients. J. Neurol. 244, 591–592 10.1007/s004150050149 9352458

[6] Merle U., Schaefer M., Ferenci P. and Stremmel W. (2007) Clinical presentation, diagnosis and long-term outcome of Wilson’s disease: a cohort study. Gut 56, 115–120 10.1136/gut.2005.087262 16709660 PMC1856673

[7] Litwin T., Gromadzka G. and Czlonkowska A. (2012) Apolipoprotein E gene (APOE) genotype in Wilson’s disease: impact on clinical presentation. Parkinsonism Relat. Disord. 18, 367–369 10.1016/j.parkreldis.2011.12.005 22221592

[8] Schiefermeier M., Kollegger H., Madl C., Polli C., Oder W., Kuhn H. (2000) The impact of apolipoprotein E genotypes on age at onset of symptoms and phenotypic expression in Wilson’s disease. Brain 123, 585–590 10.1093/brain/123.3.585 10686180

[9] Kocabay G., Tutuncu Y., Yilmaz H. and Demir K. (2009) Impact of apolipoprotein E genotypes on phenotypic expression in Turkish patients with Wilson’s disease. Scand. J. Gastroenterol. 44, 1270–1271 10.1080/00365520903225908 19722128

[10] Bu G. (2009) Apolipoprotein E and its receptors in Alzheimer’s disease: pathways, pathogenesis and therapy. Nat. Rev. Neurosci. 10, 333–344 10.1038/nrn2620 19339974 PMC2908393

[11] Shi J., Leporé N., Gutman B.A., Thompson P.M., Baxter L.C., Caselli R.J. (2014) Alzheimer’s Disease Neuroimaging Initiative. Genetic influence of apolipoprotein E4 genotype on hippocampal morphometry: an N = 725 surface-based Alzheimer’s disease neuroimaging initiative study. Hum. Brain Mapp. 35, 3903–3918 10.1002/hbm.22447 24453132 PMC4269525

[12] Anoop S., Misra A., Meena K. and Luthra K. (2010) Apolipoprotein E polymorphism in cerebrovascular & coronary heart diseases. Indian J. Med. Res. 132, 363–378 20966513

[13] Ponsford J., McLaren A., Schonberger M., Burke R., Rudzki D., Olver J. (2011) The association between apolipoprotein E and traumatic brain injury severity and functional outcome in a rehabilitation sample. J. Neurotrauma 28, 1683–1692 10.1089/neu.2010.1623 21651315

[14] Olivecrona M., Wildemyr Z. and Koskinen L.O. (2010) The apolipoprotein E epsilon4 allele and outcome in severe traumatic brain injury treated by an intracranial pressure-targeted therapy. J. Neurosurg. 112, 1113–1119 10.3171/2009.8.JNS09636 19747047

[15] Welsh-Bohmer K.A., Gearing M., Saunders A.M., Roses A.D. and Mirra S. (1997) Apolipoprotein E genotypes in a neuropathological series from the Consortium to Establish a Registry for Alzheimer’s Disease. Ann. Neurol. 42, 319–325 10.1002/ana.410420308 9307253

[16] Pedersen W.A., Chan S.L. and Mattson M.P. (2000) A mechanism for the neuroprotective effect of apolipoprotein E: isoform-specific modification by the lipid peroxidation product 4-hydroxynonenal. J. Neurochem. 74, 1426–1433 10.1046/j.1471-4159.2000.0741426.x 10737598

[17] Sayad A., Noruzinia M., Zamani M., Harirchian M.H. and Kazemnejad A. (2014) Association study of cathepsin d gene polymorphism in Iranian patients with sporadic late-onset Alzheimer’s disease. Dement. Geriatr. Cogn. Disord. 37, 257–264 10.1159/000347128 24281128

[18] Jofre-Monseny L., Minihane A.M. and Rimbach G. (2008) Impact of apoE genotype on oxidative stress, inflammation and disease risk. Mol. Nutr. Food Res. 52, 131–145 10.1002/mnfr.200700322 18203129

[19] Anoop M., Issac A., Mathew T., Philip S., Kareem N.A., Unnikrishnan R. (2010) Genetic characterization of dengue virus serotypes causing concurrent infection in an outbreak in Ernakulam, Kerala, South India. Indian J. Exp. Biol. 48, 849–857 21341545

[20] Chen Y., Li B., Zhao R.R., Zhang H.F., Zhen C. and Guo L. (2015) Increased sensitivity of apolipoprotein E knockout mice to copper-induced oxidative injury to the liver. Biochem. Biophys. Res. Commun. 459, 529–533 10.1016/j.bbrc.2015.02.143 25749341

[21] Cerpa W., Varela-Nallar L., Reyes A.E., Minniti A.N. and Inestrosa N.C. (2005) Is there a role for copper in neurodegenerative diseases? Mol. Aspects Med. 26, 405–420 10.1016/j.mam.2005.07.011 16112188

[22] Lu H. and Li C. (2006) General and highly efficient synthesis of 2-alkylideneazetidines and beta-lactams via copper-catalyzed intramolecular N-vinylation. Org. Lett. 8, 5365–5367 10.1021/ol062274i 17078719

[23] Li W., Luo H. and Zha L. (2008) Establishment of mouse model of liver damage induced by excessive copper. J. Trop. Med. 12, 007

[24] Lutsenko S. (2008) Atp7b-/- mice as a model for studies of Wilson’s disease. Biochem. Soc. Trans 36, 1233–1238 19021531 10.1042/BST0361233

[25] Huster D., Finegold M.J., Morgan C.T., Burkhead J.L., Nixon R., Vanderwerf S.M. (2006) Consequences of copper accumulation in the livers of the Atp7b-/- (Wilson disease gene) knockout mice. Am. J. Pathol. 168, 423–434 10.2353/ajpath.2006.050312 16436657 PMC1606493

[26] Methia N., Andre P., Hafezi-Moghadam A., Economopoulos M., Thomas K.L. and Wagner D.D. (2001) ApoE deficiency compromises the blood brain barrier especially after injury. Mol. Med. 7, 810–815 11844869 PMC1950012

[27] Reference deleted

